# Commonly used Hardy–Weinberg equilibrium filtering schemes impact population structure inferences using RADseq data

**DOI:** 10.1111/1755-0998.13646

**Published:** 2022-06-05

**Authors:** William S. Pearman, Lara Urban, Alana Alexander

**Affiliations:** ^1^ Department of Marine Science University of Otago Dunedin New Zealand; ^2^ Department of Anatomy University of Otago Dunedin New Zealand

**Keywords:** Hardy–Weinberg, population genetics, population genomics, RADseq, reduced representation sequencing

## Abstract

Reduced representation sequencing (RRS) is a widely used method to assay the diversity of genetic loci across the genome of an organism. The dominant class of RRS approaches assay loci associated with restriction sites within the genome (restriction site associated DNA sequencing, or RADseq). RADseq is frequently applied to non‐model organisms since it enables population genetic studies without relying on well‐characterized reference genomes. However, RADseq requires the use of many bioinformatic filters to ensure the quality of genotyping calls. These filters can have direct impacts on population genetic inference, and therefore require careful consideration. One widely used filtering approach is the removal of loci that do not conform to expectations of Hardy–Weinberg equilibrium (HWE). Despite being widely used, we show that this filtering approach is rarely described in sufficient detail to enable replication. Furthermore, through analyses of in silico and empirical data sets we show that some of the most widely used HWE filtering approaches dramatically impact inference of population structure. In particular, the removal of loci exhibiting departures from HWE after pooling across samples significantly reduces the degree of inferred population structure within a data set (despite this approach being widely used). Based on these results, we provide recommendations for best practice regarding the implementation of HWE filtering for RADseq data sets.

## 1 INTRODUCTION

Reduced representation sequencing (RRS) is a population genomic approach that enables assaying of a reduced set of genetic loci across the genome of an organism. There are many reduced representation sequencing approaches, some of which assay loci associated with restriction sites within the genome, including approaches such as genotyping‐by‐sequencing (GBS), restriction site‐associated DNA sequencing (RADseq), double digest RADseq (ddRADseq), DArTSeq, and hybridization of RAD probes (hyRAD) (see Andrews et al., 2016 for a discussion and summary of these methods). These approaches are an efficient and, in comparison with whole genome sequencing (WGS), cost‐efficient method for generating population genomic data sets, often with a focus on inferring population structure of non‐model organisms. The uniting feature of these different approaches is utilizing restriction sites in an attempt to assess genome‐wide diversity while not having to sequence the complete genome. For the remainder of this study, we group these various approaches under the umbrella term of “RADseq”.

The application of RADseq, particularly to non‐model organisms, however, can pose challenges. First, RADseq can be affected by allelic dropout, the failure to identify an allele due to the loss of a restriction site which leads to missing data for that “null” allele and therefore an apparent reduction in heterozygosity in samples (Cooke et al., 2016). Null alleles are not unique to RADseq data: for example, PCR profiling of microsatellites can manifest a null allele when a mutation in the primer‐binding site adjourning the satellite sequence inhibits amplification of that allele (Pemberton et al., 1995). There is a rich literature dedicated to the various issues such null alleles cause, including difficulties in parentage assignment, inflating population‐level estimates of inbreeding, reducing the success of assignment tests, and inflating estimates of population differentiation (Carlsson, 2008; Chapuis & Estoup, 2007; DeWoody et al., 2006; Pemberton et al., 1995). Furthermore, the inferences drawn from RADseq data originating from non‐model species often depend on the availability of a reference genome of the species of interest or a closely related one (Galla et al., 2019). While a reference genome is not essential for conducting analyses based on RADseq data sets, de novo assembly without a reference can result in more misassembled genetic loci (LaCava et al., 2020). However, as RADseq typically produces a large amount of data, bioinformatic filtering approaches can be leveraged to adjust for the potential biases of RADseq approaches.

The application of such filters help to normalize RADseq data across experiments, and to check if the data is consistent with the assumptions made by downstream analyses (O'Leary et al., 2018). For population structure inference in non‐model species (Choquet et al., 2019), downstream analyses often make assumptions about factors such as the population size (i.e., very large), the sampling scheme (i.e., randomized sampling), and the species in question (i.e., diploid). Ordination techniques such as principal component analysis (PCA) are therefore often used for preliminary analysis of RADseq data since they do not rely on these assumptions, however, they lack the translation to population parameters that are offered by parametric approaches such as admixture analyses or F‐statistics (Falush et al., 2003; Wright, 1943).

One commonly used admixture approach is STRUCTURE, a widely used tool for identifying distinct genetic groups in population genetic data, and for subsequently analysing the degree of admixture within and between individuals (Falush et al., 2003; Porras‐Hurtado et al., 2013). STRUCTURE iteratively clusters individuals into groups in order to minimize the Hardy–Weinberg disequilibrium (HWD) within groups while maximizing it between groups (Pritchard et al., 2010). Thus, STRUCTURE makes explicit assumptions about the relationship between HWD and genetic structure within groups.

F‐statistics are frequently used to infer the degree of genetic structure within predefined groups based on observed heterozygosity relative to expected heterozygosity. Population structure is typically measured using *F*_ST_, which is defined as the relative reduction in heterozygosity due to partitioning the total data set into putative populations (Whitlock, 2011; Wright, 1943). Accurate a priori delineation of groups or “populations” is essential for leveraging *F*_ST_ to characterize population structure (De Meeûs, 2018). *F*_ST_ can further be influenced by independent factors that impact the heterozygosity of individual single nucleotide polymorphisms (SNPs) (such as natural selection or technological artefacts including null alleles; De Meeûs, 2018; Meirmans & Hedrick, 2011; Whitlock, 2011).

The assumptions of the various methods highlighted here reinforce the need for appropriate bioinformatic filtering approaches when inferring population structure from RADseq data. Filtering approaches can substantially influence the inference of genetic structure, especially when filters disproportionately affect potentially informative loci (Graham et al., 2020; Shafer et al., 2017). Linck and Battey (2019) showed that minor allele frequency (MAF) filtering of data sets may be problematic since it alters the site frequency spectrum (SFS) across loci according to their rate of missingness. Additional recent studies have revealed that both variant call rate and MAF can affect population genetic inferences and genotype‐environment association studies (Ahrens et al., 2021; Selechnik et al., 2020). In Table 1, we summarize filtering approaches that are commonly applied to RADseq data, the reasons for their usage, and how they can affect population genetic inference.

**TABLE 1:** Description of commonly used filtering approaches in the analysis of RADseq data (“filter”), the reason for their usage (“usage”), and how they impact population genomic inference (“impact”)

Filter	Usage	Impact	Reference
Hardy–Weinberg equilibrium (HWE)	Removes loci under selection Removes library and sequencing artefacts	Unknown	Gruber et al. (2018), Sethuraman et al. (2019), Waples (2015)
Linkage within loci	Mitigates effects of nonindependence of single nucleotide polymorphisms (SNPs) by removing physically linked SNPs.	Reduces false signals of population structure Necessary for STRUCTURE (if LD correction is not used)	O'Leary et al. (2018)
Locus level diversity	Loci with high SNP density (i.e., many SNPs within a locus) may be the result of polyploidy	Can remove putative paralogous loci	Hohenlohe et al. (2011), Mastretta‐Yanes et al. (2015)
Minor allele frequency (MAF)/count (MAC)	Identification of genotyping errors	Can remove informative loci if not applied carefully MAF will affect loci differently based on missingness Removes genotyping errors	Linck and Battey (2019), O'Leary et al. (2018)
Variant call rate	Ensures SNP panel is well represented across individuals	Can dramatically reduce number of loci Helps ensure samples are comparable	O'Leary et al. (2018)

The removal of genetic loci exhibiting departures from Hardy–Weinberg equilibrium (HWE) is a commonly applied filter (Waples, 2015). HWE describes the state of an ideal population in the absence of evolutionary forces, where allele frequencies are predictable since they remain constant across generations (Garnier‐Géré & Chikhi, 2013).

The identification of genetic loci departing from HWE is often used to remove loci subject to genotyping errors such as null alleles (Hendricks et al., 2018) and loci that are potentially under selection (Lachance, 2009; Wang et al., 2005). The removal of genotyping errors is, in general, beneficial for downstream analyses, while the removal of loci under selection may be required for analyses that assume neutrality of loci. However, many other factors can cause departures from HWE, especially since the assumptions of HWE are rarely met in real biological populations (Waples, 2015), and therefore the removal of loci out of HWE may have substantial effects on population genetic inferences.

The, arguably, most obvious other factor that can cause departures from HWE is the Wahlund effect due to the inadvertent pooling of multiple populations (De Meeûs, 2018). Under the Wahlund effect, expected heterozygosity of loci is, on average, reduced relative to observed heterozygosity, with the reduction in heterozygosity proportional to *F*_ST_. Excessive deviation from HWE heterozygosity expectations can also arise from repetitive genomic elements (Hohenlohe et al., 2011). Other scenarios that lead to HWE departure, frequently observed in real populations, include overlapping generations, non‐panmictic reproduction, non‐diploidy, and very small population sizes. Genotype/single nucleotide polymorphism (SNP) calling approaches represent further potential sources of departure from HWE: Genotype calling can be sensitive to sequencing depth, and to the number of mismatches allowed to call a variant, both of which can lead to a reduction in heterozygosity and in turn lead to HWE departures (Cumer et al., 2021).

While the impact of such factors is often minor, genetic inferences for species that have many potential causes of HWE departures (such as endangered species) might be heavily impacted by decisions around HWE‐based filtering. Specifically, when conservation decisions are based on genetic inferences that utilize HWE filtering, it is essential to ensure that this is done appropriately to aid in the management of already vulnerable populations.

**FIGURE 1:**
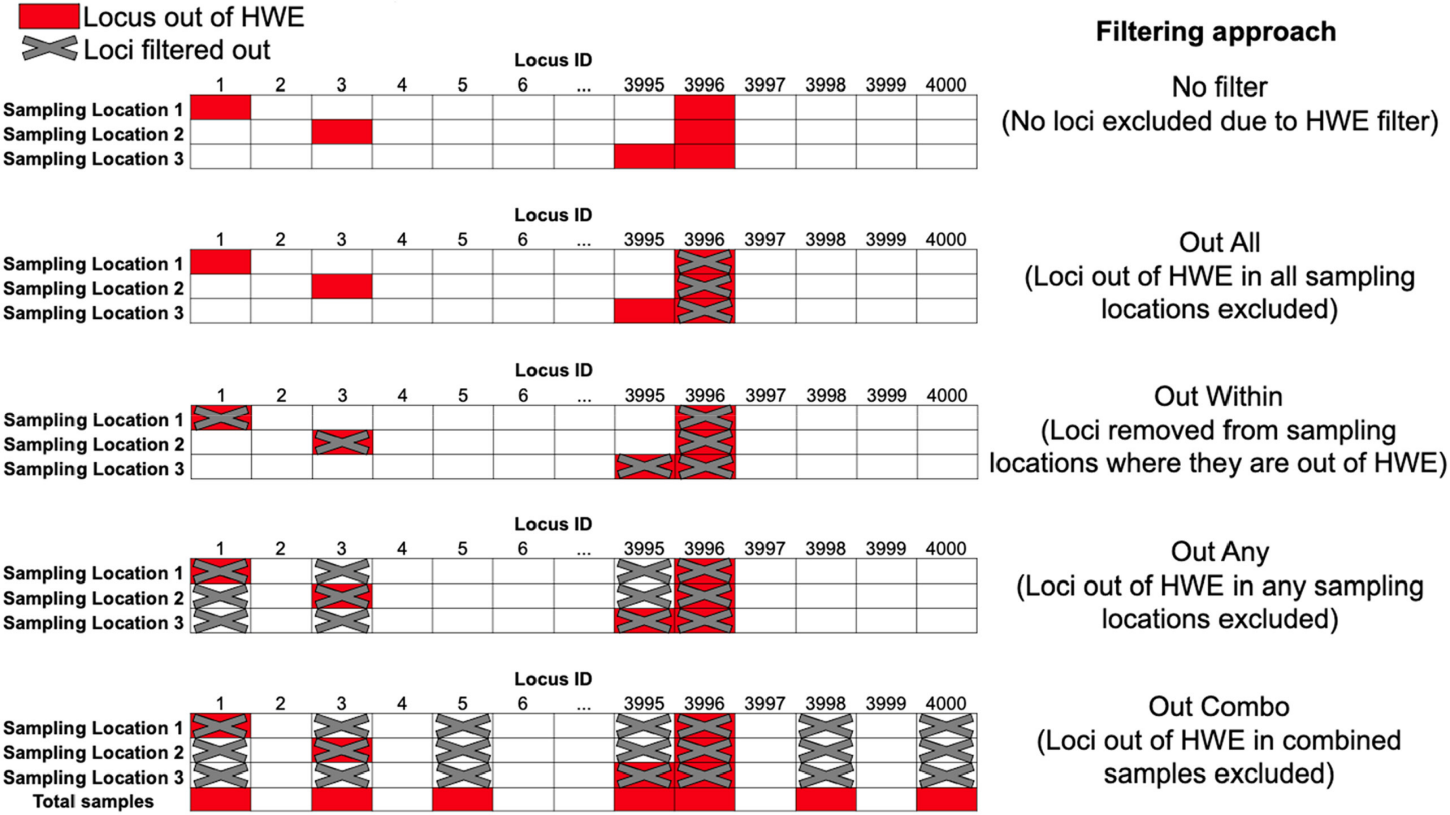
Five potential Hardy–Weinberg equilibrium (HWE) filtering options (loci removed indicated by grey crosses). In the case of “no filter”, no loci are removed, even if they exhibit departures from HWE. In the case of “out all”, loci are removed if they exhibit departures from HWE in all sampling locations. “Out some” can be considered a subset of “out all”, where loci are removed if they are out of HWE in a certain proportion of populations. In “out within”, loci are removed only from the populations in which they depart from HWE. In “out any”, loci are removed if they exhibit departures from HWE in any of the sampling locations and, finally, in “out combo”, loci are removed if they exhibit HWE departures when sampling locations are grouped together

The question of when, and if so how, a genomic data set should be filtered for departure from HWE is a difficult one. Sample stratification has to be taken into account; genetic loci that depart from HWE can be filtered in various ways (Figure 1): No loci removed based on HWE departures (“No Filter”), loci removed if they exhibit departures in any sampling location (“Out Any”), loci removed if they exhibit departures from HWE in all sampling locations (or a certain proportion of sampling locations) (“Out All”, “Out Some”), loci removed if they exhibit departures across sampling locations (“Out Combo”). Finally, loci can be removed from the populations in which they exhibit deviations from HWE (“Out Within”) (Figure 1).

The “Out Combo” approach removes genetic loci that depart from HWE across the entire genomic data set. This filtering scheme will have a substantial impact on downstream analyses since loci that are strongly informative for population structure are likely to be removed by this filter due to the differences in allele frequencies between populations leading to these loci to being out of HWE when analysed at the total data set level. However, applying “No Filter” could lead to the retention of genotyping errors or of genetic loci under selection which might be problematic in downstream analyses. A potentially equally extreme filter would be “Out Any”, in which loci are discarded if they exhibit deviations from HWE in any population. Middle ground approaches between these two extremes may be “Out All”, or “Out Within”. In the “Out All” (or “Out Some”) approach, only loci that depart from HWE in all (or some) populations would be removed, that is, the loci that are most likely to be problematic. In the “Out Within” approach, loci are only removed from populations where they depart from HWE—this would maximize the number of loci available for pairwise comparisons and thus retain the most information possible. However, these latter three approaches (“Out Any”, “Out All”, and “Out Within”) require knowledge of the underlying population structure in order to correctly define populations for assaying patterns of HWE. In the absence of prior knowledge, studies often assume sampling locations to be a proxy for genetic populations. While this assumption might not be problematic in the case of pronounced population structure, conflating sampling location with genetic populations in the case of subtle population structure could be problematic. This is because the application of HWE filters might inflate divergence estimates between sampling locations if they do not accurately map to the underlying population structure (i.e., where different sampling locations represent the same underlying panmictic population). This inflation may occur if loci that discriminate “true” populations were removed through HWE filters, and loci that discriminate sampling locations were retained. This would erroneously reinforce the a priori hypothesis that sampling locations reflect underlying genetic populations. This “over‐splitting” of populations can be as problematic in a conservation setting as the previously discussed “over‐lumping” of populations (i.e., Wahlund effects) in terms of implementing management recommendations.

Despite the potentially substantial impact of HWE‐based filtering approaches, they are frequently misused or their application is not reported at all (Sethuraman et al., 2019). While it has been suggested that HWE filtering is often inadequately described and inappropriately applied (Gruber et al., 2018; Waples, 2015), this has not yet been systematically assessed within the field of RADseq‐based population genomic research (Table 1). For example, many widely used filtering tools such as VCFtools (Danecek et al., 2011), plink (Chang et al., 2015), and pegas (Paradis, 2010) calculate HWE departures directly from genetic data rather than utilizing a population mapping file (i.e., the default behaviour would be “Out Combo”, subject to the impact of the Wahlund effect). This default behaviour might be desirable when studying a single population, as is often the case in large‐scale human genomic studies, but it could be problematic in studies comprising many populations for the reasons outlined above.

Here, we first review the common approaches for HWE filtering currently used in the RADseq literature, and then systematically explore the effect of different HWE filtering approaches with the help of simulations and empirical biological data sets across a wide range of realistic levels of population structure. We hypothesise that HWE filtering will have a substantial effect, especially on marginally or nons‐tructured populations. Specifically, we hypothesise that the removal of genetic loci that depart from HWE across populations will reduce estimated population structure, whereas the removal of genetic loci that depart from HWE in any population will increase estimated population structure and divergence by reducing the impact of “noisy” loci resulting from methodological artefacts (e.g., variant calling, null alleles). Finally, we hypothesise that HWE filtering schemes that consider population strata will reinforce the a priori sample groupings when genetic populations are conflated with sampling locations.

## 2 MATERIALS AND METHODS

### 2.1 Literature review

We conducted a literature review for RADseq‐based population genomic research using the Web Of Science (see Supporting Information material for specific search terms). From the initial results, we selected studies that contained any of the following terms “Hardy”, “Weinberg”, “HWE” or “Hardy–Weinberg”, and excluded those that met any of the following criteria:

1. Described a new panel of SNPs; these studies mostly describe a very small panel of genetic variants.
2. Studied a single population; studying a single population means that HWE filtering will not have an impact on population structure inference.
3. Focused on human populations; we excluded human data sets to avoid ethical concerns around demarcating human populations and the comparatively rare use of RADseq for humans compared to WGS.
4. Consisted of transcriptome‐ or RNA‐derived genetic variants; these variants are likely to display departures from HWE since they are transcriptionally expressed and therefore more likely to be under selection.
5. Did not explicitly discuss HWE filtering; we were not able to discern if these studies had not applied any filtering or had just not mentioned it. Furthermore, it was difficult to ascertain whether this filter was overlooked or intentionally avoided and would bring the scope of the literature review beyond what was manageable.
6. Was not based on RADseq data; we focused on RADseq data since allelic dropout can be a substantial source of HWE departures, and RADseq is currently one of the predominant RRS approaches for non‐model organism population genetics.

The remaining studies were classified into one of the seven categories described in Table 2 (note that “No Filter” probably underestimates the number of studies that do not utilize Hardy–Weinberg filtering, as studies that do not discuss this would not be included in our search results—as we explicitly searched for Hardy–Weinberg associated studies).

**TABLE 2:** Description of categories used to group scientific studies based on their Hardy–Weinberg filtering approaches, “HWE Out Within” was not observed among these studies

Category	Definition
HWE Out All	Loci were excluded if they were out of HWE in every sample location
HWE Out Any	Loci were excluded if they were out of HWE in at least one of the sampling locations
HWE Out Some	Loci were excluded if they were out of HWE in at least a specific absolute number or relative proportion of the locations, but not in all locations
HWE Out Combo	Loci were excluded if they were out of HWE when samples were pooled across all locations
No Filter	The study explicitly mentions that no loci were removed due to HWE filtering
Unspecified	HWE filtering was used, but no specific filtering approach was described
Mix	A combination of these categories was used

### 2.2 Simulated data

To investigate the impact of HWE filtering on inference of population structure, we used both simulated and empirical data sets. For all simulations, we used the SLiM forward genetic simulation framework (Haller & Messer, 2016; Messer, 2013) with 10 replicates with randomly generated seeds for each simulation. We simulated a random genome approximately 104 megabases in length. We conducted two sets of simulations, with either a recombination rate of 1 × 10^−4^ (high recombination/low linkage) or of 1 × 10^−8^ (low recombination/high linkage) in combination with the “pseudochromosomes” option in SLiM to enable independent simulation of 13 autosomal chromosomes (Waples et al., 2021). We assumed a sexually reproducing diploid organism. We chose an arbitrary but realistic mutation rate of 10^−8^, and an effective population size of 1000. Age‐related mortality was implemented with maximum mortality at age seven, with density‐dependent survival ensuring fluctuation of the population size around the effective population size.

A single population was created which evolved for 135,000 generations (which was three times the number of generations that the initial population took to reach coalescence, namely approximately 45,000 generations), followed by divergence into 12 separate populations with an initial census population size of 80. These populations then evolved for another 15,000 generations with constant migration between adjacent populations (Figure S1). During this period, populations expanded to an effective population size of 1000. Differing migration rates in each scenario adjusted the degree of population structure, with the “Marginal” population structure migration rate at 0.1 (i.e., 0.1 or 10% of a population was transferred to the adjacent population/s in each generation, e.g., population 5 received 10% of population 4 and 10% of population 6), “Low” population structure migration rate at 0.01, “High” population structure migration rate at 0.001, and “Extreme” population structure migration rate at 0.0001. Migration was deterministic, and the number of migrant individuals from each population drawn using a Poisson distribution with a lambda equal to the product of the population size and the migration rate. At generation 150,000, 30 individuals were sampled randomly from every other adjacent population, resulting in a total of 180 individuals being sampled from populations 1, 3, 5, 7, 9, and 11 (Figure S1).

Panmictic scenarios were created similarly, however rather than dividing the population among 12 subpopulations at generation 135,000 the population underwent a bottleneck to 1000 individuals and grew until an *N*_e_ of 12,000 was reached. After an additional 15,000 generations, 180 individuals were randomly sampled and carried forth into further analyses. For these panmictic scenarios, we evenly split the 180 individuals into six “pseudopopulations”, in order to understand the influence of incorrect sample divisions on population genetic inference.

The resulting VCFs were processed by the program RADinitio, which simulates the RADseq process, including restriction enzyme digest and sources of error (e.g., sequencing error, variation in read depth across alleles) (Rivera‐Colón et al., 2021). We used PstI as a restriction enzyme, set mean coverage at 10×, and simulated nine PCR cycles, a read length of 150 bp, and a mean insert length of 350 bp with a standard deviation of 35 bp. These settings were chosen to ensure computational tractability of the thousands of simulations and enable compatibility of SLiM and RADinitio, while also choosing realistic RADseq library features. The simulated fastq reads were aligned to the reference using BWA v.0.7.17 (Li, 2013; Li & Durbin, 2009); we then used SAMtools v1.10 (Li et al., 2009) to convert the alignments to sorted bam files. SNPs were called using a reference‐guided Stacks v2.53 workflow (Rochette et al., 2019). We called Stacks via ref_map.pl using default options: 0.05 as the significance level for calling variant sites (var‐alpha) and genotypes (gt‐alpha), PCR duplicates were not removed, paired‐end reads and read pairing were utilized (i.e., we did not use the rm‐pcr‐duplicates, ignore‐pe‐reads, and unpaired flags), the minimum percentage of individuals in a population required to output a locus was zero (−‐min‐samples‐per‐pop/−r), and the minimum number of populations a locus had to be present in was one (−‐min‐populations/−p). We then used the populations module of Stacks to write one random SNP from each locus to a VCF file as input for downstream analyses (i.e., using the write‐random‐snp and VCF flags).

### 2.3 Empirical data

In order to test the generality of our simulations against empirical data sets, we selected three publicly available data sets as they represented a range of organisms, with a range of population structure: A diversity arrays technology sequencing (DArTseq) data set of a New Zealand isopod (*Isocladus armatus*) (Pearman et al., 2020), and two RADseq data sets of the New Zealand fur seal (*Arctocephalus forsteri*) (Dussex et al., 2018) and the Plains zebra (*Equus quagga*) (Larison et al., 2021). For the isopod data set, the DArTseq genotypes were provided by diversityarrays, who generated them using their proprietary SNP calling software with a *de novo* assembly (SRA: PRJNA643849, https://osf.io/kjxbm/). For the other two data sets, a Stacks workflow similar to the in silico analyses was used to generate the SNP genotypes. SRA data (New Zealand fur seal: SRP125920, single‐end data; and zebra: SRP288329, paired‐end data) was obtained (using prefetch) and converted to fastq (using fastq‐dump) with sratoolkit v2.9.6 (Leinonen et al., 2011). Metadata associated with these data sets (Dussex et al., 2018; Larison et al., 2021) was used to generate popmap files. Congeneric genomes were used as references, namely Antarctic fur seal for the New Zealand fur seal analyses (GCA_900642305.1_arcGaz3_genomic: Humble et al., 2018) and horse for the zebra analyses (GCF_002863925.1_EquCab3.0_genomic: Kalbfleisch et al., 2018). The Stacks workflow then followed the previously described workflow for the in silico data sets.

### 2.4 SNP filtering

For both in silico and empirical data sets, we filtered data on a minor allele count of 2 (such that singletons were removed, but doubletons were retained) (Linck & Battey, 2019), call rate of 0.8 (such that sites with >20% missing data across individuals were removed) (Rochette et al., 2019), and then applied various filtering approaches for SNPs departing from HWE (Figure 1). SNPs exhibiting departures from HWE corresponding to each filtering scheme (i.e., Out Any, Out All, Out Combo, and Out Within) were identified using the function hw.test in the pegas R package (Paradis, 2010). This function uses a ꭓ^2^ test for departures from expected Hardy–Weinberg proportions. We corrected for multiple testing using a false‐discovery rate correction (*n* = 4000, based on the number of loci) (Benjamini & Hochberg, 1995), and subsequently removed SNPs exhibiting HWE departures using VCFtools.

### 2.5 Data analysis

To examine variance in our parameter estimates, we conducted binomial sampling (with replacement) from the total number of SNPs in each filtered VCF to generate 10 VCF files consisting of 4000 SNPs each. To examine population structure, we conducted principal component analysis (PCA), *F*_ST_, and STRUCTURE analyses. PCAs were conducted in R 4.02 (R Core Team, 2020), using a genotype matrix with scaled genotypes following procedures outlined in Linck and Battey (2019) in the adegenet R package (Jombart & Ahmed, 2011). PCAs were compared using the PC_ST_ metric, which represents one minus the ratio of the mean within‐population distance to total‐population distance within a PCA. Higher values of PC_ST_ are consistent with higher levels of population structure (see Linck & Battey, 2019 for an in‐depth explanation). Due to equal sample sizes among populations, the modelling of RADseq rather than array data (Bhatia et al., 2013), and its status as a common *F*_ST_ estimator, we used Weir and Cockerham's pairwise *F*_ST_ (Weir & Cockerham, 1984), calculated using the R package STaMMP (version 1.6.1), and the mean of these pairwise values was used in downstream analyses (Pembleton et al., 2013).

STRUCTURE (v 2.3.4) was run using an admixture model with no a priori information regarding population structure, using a *K* of 6 for our in silico data, or a *K* equivalent to the number of sampled populations for the empirical data sets. STRUCTURE analyses were run for 100,000 iterations, with a burnin of 50,000. While convergence is not routinely assessed in STRUCTURE analyses, we assessed this through examining the modality of the log likelihoods for each set of analyses. Pairwise comparisons of filters within each scenario were tested for significance using Mann–Whitney U tests and FDR adjustment (alpha = 0.05) in R 4.02 using rstatix (version 0.7.0) (Kassambara, 2021; R Core Team, 2020). Figures were created using the tidyverse and cowplot packages (Wickham et al., 2019; Wilke, 2020).

In order to elucidate the relationship between observed HWE deviations and a Wahlund effect, we regressed the slope of *F*_ST_ against *F*_IS_, with the expectation that a Wahlund effect would result in a positive relationship between *F*_IS_ and *F*_ST_ due to heterozygote deficiency (Waples, 2015; Zhivotovsky, 2015). For these analyses, we pooled all individuals into a single population, *F*_IS_ was calculated using hierfstat (with *F*_IS_ = 1‐*H*_O_/*H*_t_) and regressions were conducted using base *R*.

## 3 RESULTS

### 3.1 Literature review

**FIGURE 2:**
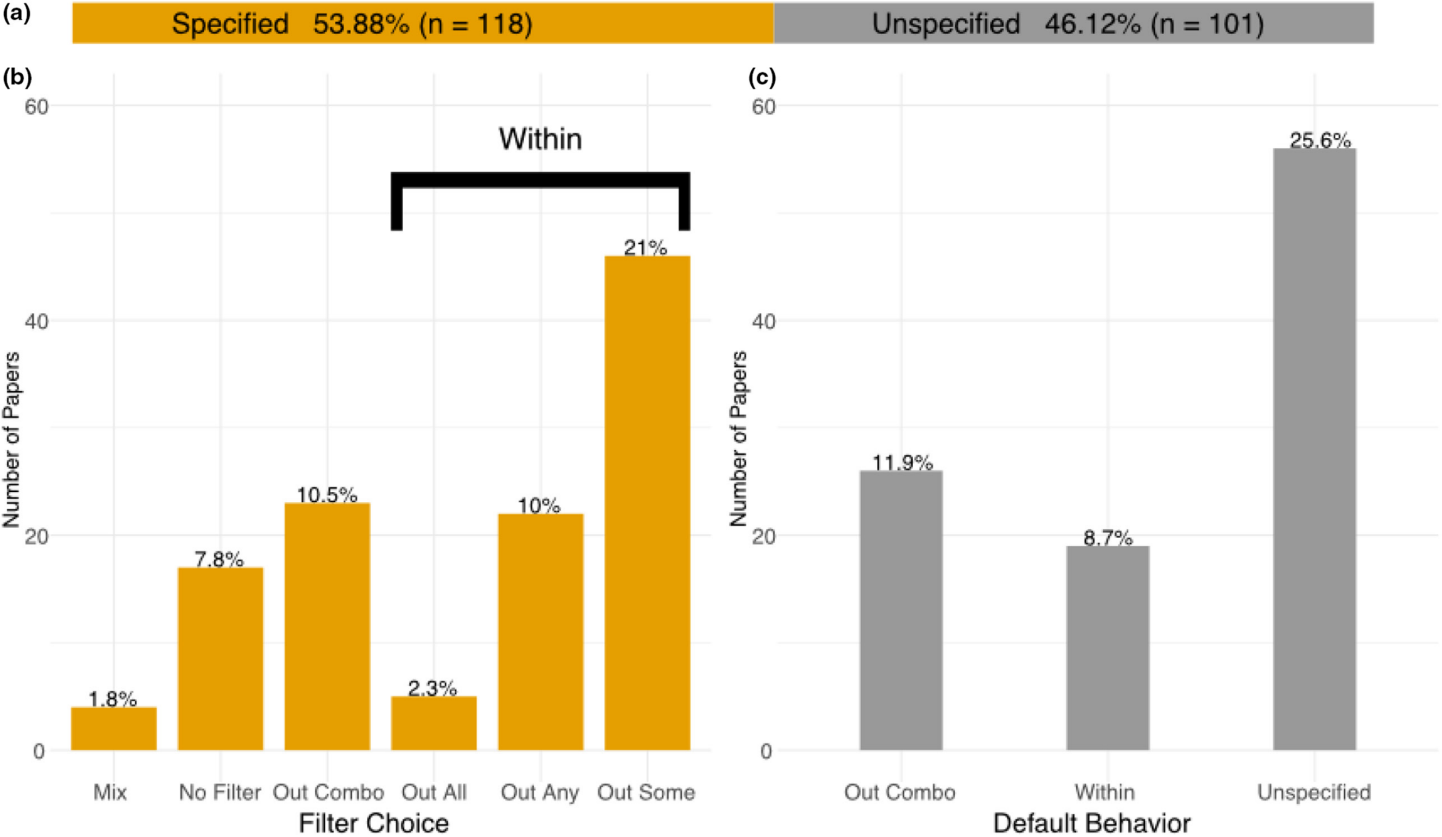
(a) Distribution of publications that specified their HWE filtering approach (orange) versus publications that did not specify the approach in sufficient detail (grey). (b) the distribution of publications that specified their HWE filtering approach across different filtering schemes: “Mix” (mix of the following filters), “no filter” (no HWE filter), “out combo” (loci removed if out of HWE across the pooled data set), “out all” (loci removed if out of HWE in each sampling location), “out any” (loci removed if out of HWE in any sampling location), and “out some” (loci removed if out of HWE in at least a certain number/relative proportion of sampling locations, but not in all locations). (c) the distribution of publications that did not specify Hardy–Weinberg filtering approach and with the default behaviour of the filtering tools used (where specified) assumed: “Out combo” (as defined above), “within” (the paper specified that they used population information for HWE filtering, but not specifically whether this was “out all”, “out any”, or “out some”) and “unspecified” (the paper did not specify the tool)

Our literature review of 219 scientific publications concerning HWE filtering of RADseq data showed that 53.88% of the publications (*n* = 118) specified their HWE filtering approach (Figure 2a). Overall, 21% of the publications used some intermediate threshold (“Out Some”) to filter SNPs departing from HWE, 10.5% used “Out Combo”, 10% used “Out Any”, 7.8% explicitly chose not to filter for HWE departure and outlined their reasons, and 2.3% used “Out All” (Figure 2b; see Table 2 for definition of filtering approaches). The remaining 101 publications (46.12% of all publications) did not specify the HWE filtering approach in sufficient detail (Figure 2a): 45 publications (20.6% of all publications) specified only the filtering tool they used, whereas the remaining publications (25.6% of all publications) did not specify any information (“Unspecified”; Figure 2c). If the default behaviour of the specified filtering tools is assumed, another 11.9% of all publications (*n* = 26) used “Out Combo” (Figure 2c). Overall, this means that at least 22% of the publications that filtered for departure from HWE have most likely used the “Out Combo” approach, but we expect this proportion to be even higher due to the large proportion of unspecified publications. Finally, some publications (8.7%, *n* = 19) used filtering tools that explicitly consider population stratification in HWE calculations such as Arlequin (Excoffier et al., 2005), or Genepop (Rousset, 2008), but the publications did not report the exact filtering approach (“Within”, Figure 2c).

### 3.2 In silico data analysis

**FIGURE 3:**
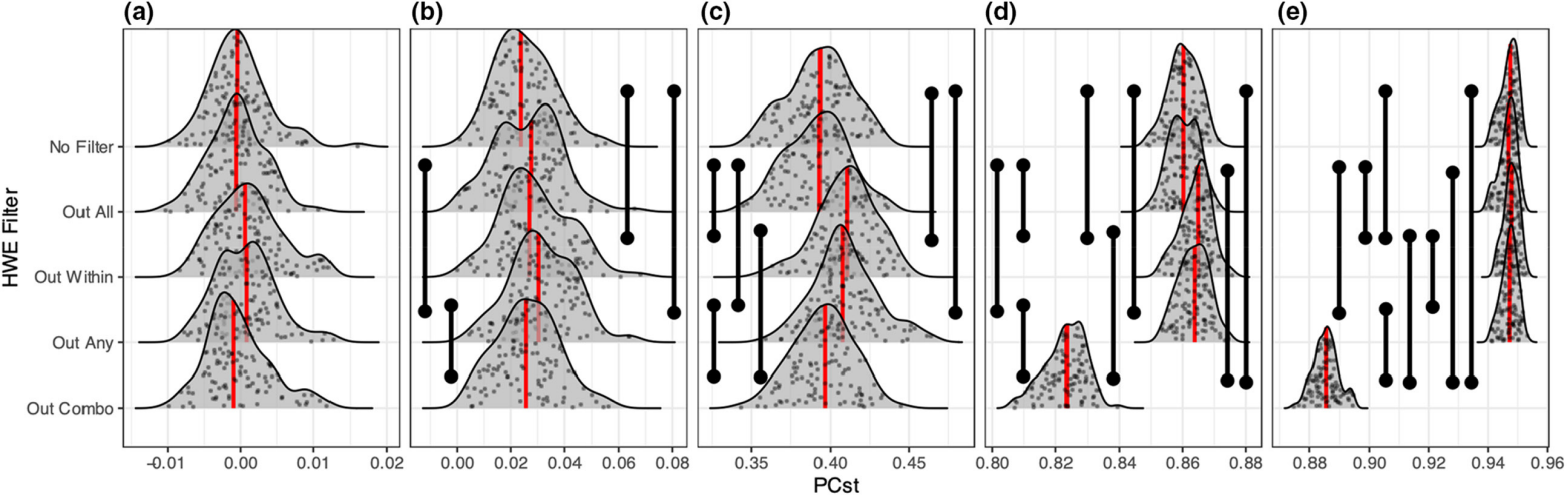
Distributions of low linkage PC_ST_ across HWE filtering approaches and degrees of population structure. (a) Represents no population structure (single panmictic population), (b) represents marginal population structure (i.e., high migration, M = 0.1), (c) represents low population structure (*M* = 0.01), (d) represents high population structure (*M* = 0.001), and (e) represents extreme population structure (i.e., low migration, *M* = 0.0001). Red lines indicate median values, black vertical bars indicate statistically significant comparisons (Mann–Whitney *U* tests, FDR adjustment). *Y*‐axis values are arbitrary in order to display the raw data

**FIGURE 4:**
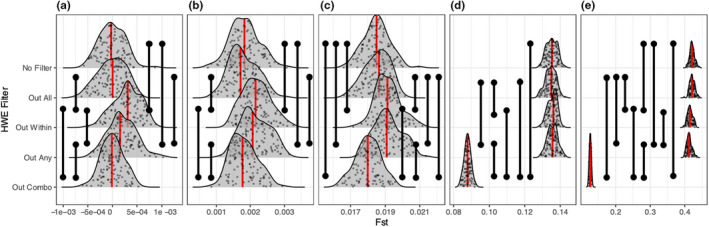
Distributions of low linkage inferred *F*_ST_ across HWE filtering approaches and degrees of population structure. (a) Represents no population structure (single panmictic population), (b) represents marginal population structure (i.e., high migration, *M* = 0.1), (c) represents low population structure (*M* = 0.01), (d) is high population structure (*M* = 0.001), and (e) represents extreme population structure (i.e., low migration, *M* = 0.0001). Red lines indicate median values, black vertical bars indicate statistically significant comparisons (Mann–Whitney *U* tests, FDR adjustment). *Y*‐axis values are arbitrary in order to display the raw data

We found that the two linkage levels we included in our simulations lead to minor differences in *F*_ST_ and PCst, with high linkage simulations having marginally higher structure (Figures 3 and 4 and Figures S2 and S3). Despite these minor differences, the relative effects of each filtering scheme tended to be consistent across the two linkage levels. Therefore, here we focus on the low linkage results, with the high linkage results available in the Supporting Information materials.

Measurements of population stratification extracted from PCAs (PC_ST_) were affected most strongly by the “Out Any”, “Out Within”, and the “Out Combo” filters. For “Out Any” and “Out Within”, we observed marginal increases in PC_ST_ relative to other filters, and these differences were frequently statistically significant with the exception of the panmictic scenario (Figure 3a). In this scenario the increase was not significant for the low linkage analysis; however, it was significant relative to other filters in the high linkage analysis (Figure S2).

The effect of “Out Combo” became apparent with increasing population structure, reducing PC_ST_ estimates in comparison with other filtering approaches at high and extreme levels of population structure, but with relatively little effect at lower levels (Figure 3). The “No Filter” and “Out All” approaches delivered qualitatively similar PC_ST_ estimates. This indicates that the “Out Combo” filter reduces estimated population structure evident in a PCA in relation to the other filtering schemes, while “Out Any” and “Out Within” tended to increase estimated structure.

We similarly observed an increasingly strong reduction of inferred *F*_ST_ with increasing levels of population structure when utilizing the “Out Combo” filtering approach (Figure 4, Figure S3). While “Out All” and “No Filter” consistently delivered similar *F*_ST_ estimates to each other, we found that “Out Any” and “Out Within” led to larger inferred *F*_ST_ values that were similar to each other, with the exception of extreme population structure where *F*_ST_ was slightly (but significantly) reduced for these filtering approaches. In the no population structure scenario (Figure 4a), “Out Within” and “Out Any” led to larger *F*_ST_ values than other filters, indicating the introduction of spurious population structure (Figure 4, Figure S3).

*Post hoc* calculations of statistical power (Serdar et al., 2021) suggested we had extremely high power to detect differences in *F*_ST_ (~100%) between at least one pair of filter regimes within each scenario/linkage combination (data not shown). This suggests the combination of sample sizes, number of replicates, and effect sizes within our data gives adequate power for the purposes of this study for example, highlighting the broad impact of different filtering schemes on apparent population structure.

**FIGURE 5:**
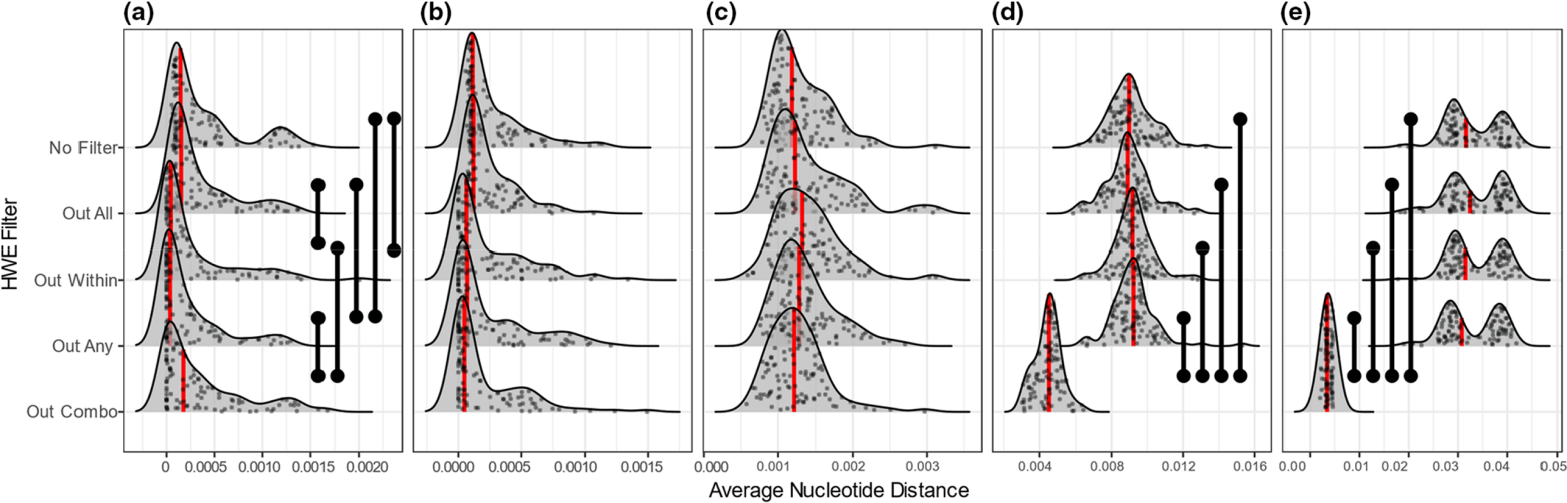
Distributions of the low linkage average nucleotide distance between inferred population clusters from STRUCTURE, across differing filtering regimes and levels of population structure. (a) Represents a panmictic scenario with no population structure. (b) Represents marginal population structure (i.e., high migration, M = 0.1), (c) represents low population structure (*M* = 0.01), (d) is high population structure (*M* = 0.001), and (e) represents extreme population structure (i.e., low migration, *M* = 0.0001). Red lines indicate median values, black vertical bars indicate statistically significant comparisons (Mann–Whitney *U* tests, FDR adjustment). *Y*‐axis values are arbitrary in order to display the raw data

For the STRUCTURE analyses, we observed that the “Out Combo” filter significantly increased the average nucleotide distance between inferred population clusters in the panmictic population structure scenarios, while also decreasing the inferred amount of structure in the high and extreme population structure scenarios (Figure 5). “Out Any” and “Out Within” lead to marginal increases in nucleotide distances for some population structure scenarios (Figure 5), however statistical significance only occurred in some of the high linkage scenarios (Figures S4). In almost all cases, we found that STRUCTURE runs appeared to reach convergence across replicates (Figures S5 and S6; also indicated by the unimodal distributions in Figure 5). The exception to this was the “extreme” population scenario, where it appeared convergence may have been impacted by the underlying simulated population structure of 12 populations. We reran this scenario with a *K* of 12, which demonstrated an increase in convergence across replicates (Figures S7 and S8).

### 3.3 Empirical data analysis

**FIGURE 6:**
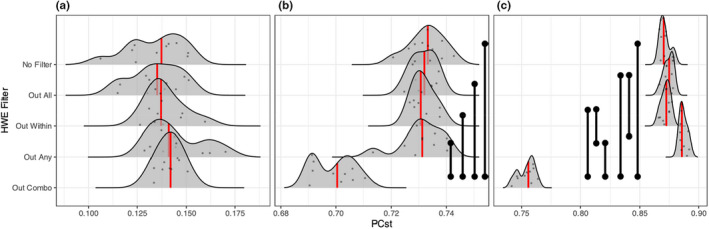
PC_ST_ distributions for empirical data sets. (a) Represents New Zealand fur seal data (*Arctocephalus forsteri*), (b) represents plains zebra (*Equus quagga*), and (c) represents a New Zealand isopod (*Isocladus armatus*). Red lines indicate the median value for each distribution, black vertical bars indicate statistically significant comparisons (Mann–Whitney *U* tests, FDR adjustment). Species ordered from low population structure (New Zealand fur seal) to high population structure (isopod). *Y*‐axis values are arbitrary in order to display the raw data

The results from the empirical data sets were generally concordant with those from the simulations. No significant differences were observed among filters for PC_ST_ in the species with the weakest population structure, the New Zealand fur seal (Figure 6a). In the species with more pronounced population structure (Plains zebra and isopod, Figures 6b–c), the “Out Combo” filter significantly reduced PC_ST_ in comparison with the other filters. “Out Any” had marginally higher estimated structure than all other filters in the isopod data set (Figure 6c).

**FIGURE 7:**
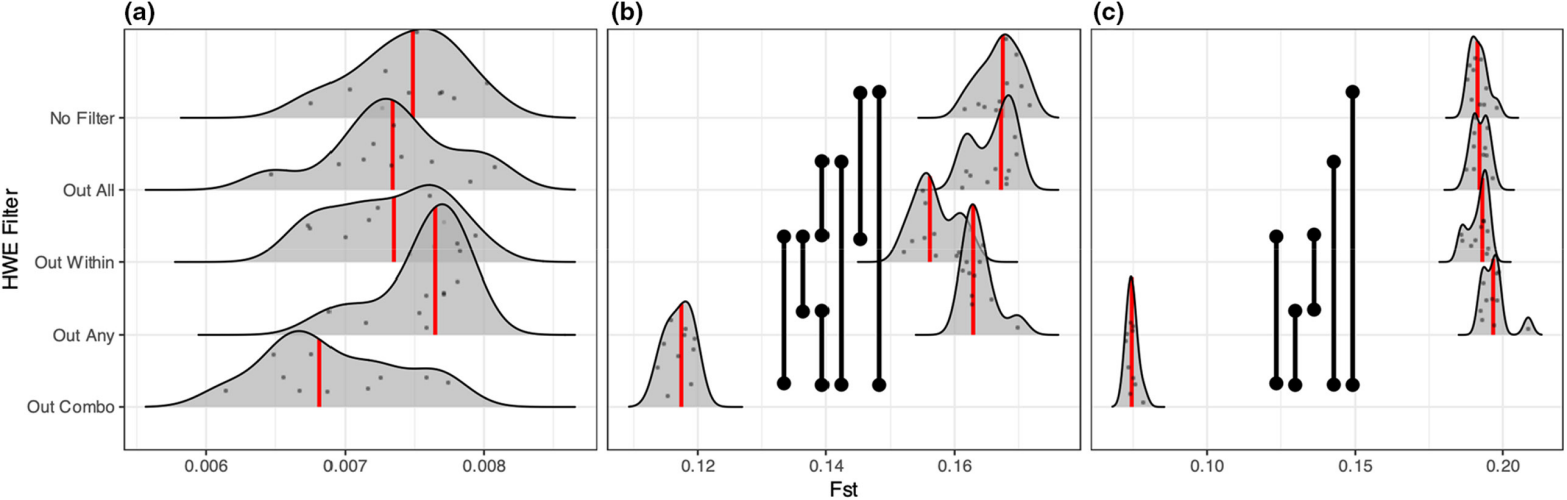
*F*_ST_ distributions for empirical data sets, (a) represents New Zealand fur seal data (*Arctocephalus forsteri*), (b) represents plains zebra (*Equus quagga*), and (c) represents a New Zealand isopod (*Isocladus armatus*). Red lines indicate the median value for each distribution, black vertical bars indicate statistically significant comparisons (Mann–Whitney *U* tests, FDR adjustment). Species ordered from low population structure (New Zealand fur seal) to high population structure (isopod). *Y*‐axis values are arbitrary in order to display the raw data

Similar results were obtained for *F*_ST_ (Figure 7)_,_ where the filtering approaches had only small impacts for the inference of population structure in the species with low population structure (New Zealand fur seal), while “Out Combo” significantly reduced *F*_ST_ estimates for the species with higher levels of population structure (Plains zebra and isopod).

**FIGURE 8:**
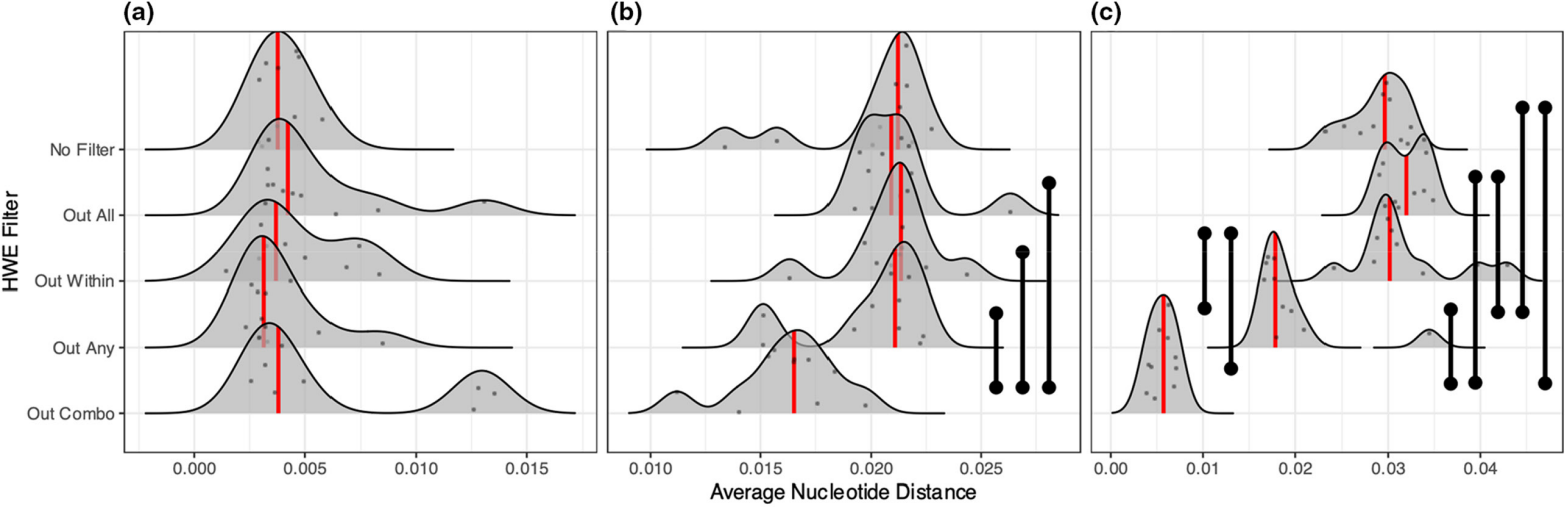
Nucleotide distance distributions for empirical data sets, (a) represents New Zealand fur seal data (*Arctocephalus forsteri*), (b) represents plains zebra (*Equus quagga*), and (c) represents a New Zealand isopod (*Isocladus armatus*). Red lines indicate the median value for each distribution, black vertical bars indicate statistically significant comparisons (Mann–Whitney *U* tests, FDR adjustment). Species ordered from low population structure (New Zealand fur seal) to high population structure (isopod). *Y*‐axis values are arbitrary in order to show the raw data

The “Out Combo” filtering approach similarly reduced the estimated nucleotide distance from STRUCTURE between clusters for zebra and isopod (the species with the most marked population structure; Figure 8). In addition, the “Out Any” filtering approach led to a significant reduction in estimated nucleotide distance in the isopod data set (Figure 8c). In all cases, STRUCTURE runs appeared to have reached convergence based on mean log likelihoods (Figure S9).

## 4 DISCUSSION

There are many good reasons to impose a filter for HWE, such as removal of loci under extreme selection, paralogues, and sequencing or library preparation artefacts. Thus, HWE filtering can be helpful in standardizing and denoising a data set. However, in this study, using both empirical and simulated data sets, we demonstrate that filtering SNPs based on HWE can have substantial impacts on population genetic inferences. In particular, we found that the “Out Combo” filtering approach, where loci that depart from HWE across all pooled samples are removed, significantly reduces the amount of inferred population structure relative to “No Filter” or other filtering approaches. This occurs because this filter leads to the inadvertent introduction of a Wahlund effect by not considering any existing population structure, with loci important for delineating population structure being removed by the HWE filter. Despite the strong impact of HWE filtering, our literature review shows that the vast majority of scientific publications that report filtering for HWE do not include sufficient detail to allow replication of this aspect of their analyses. This often occurs because only the filtering tool or significance threshold is reported, while population stratification for filtering is not defined. When default behaviour of filtering tools is assumed, up to 50% of publications may be misapplying HWE filtering (Figure 2), by using the “Out Combo” filtering approach. Some commonly used filtering tools such as VCFtools and plink do not consider population structure when calculating deviations from HWE, and therefore the reliance on default settings may lead to the removal of the very loci that are informative for population structure. Importantly, even the implementation of an extremely conservative significance level for identifying “problematic” loci will not solve the issues of the “Out Combo” filtering approach, as an extreme Wahlund effect will be observed in instances of extreme population structure—which would naturally draw loci closer to even stringent significance levels.

We hypothesized that (1) use of an “Out Combo” filter would substantially reduce inferred population structure, and (2) that the use of an “Out Any” filter would lead to an increase in inferred population structure. Consistent with these hypotheses we found that (1) filtering across populations (“Out Combo”) had the greatest effect, substantially reducing inferred population structure, and (2) filtering loci that were out of HWE in any population (“Out Any”) had a marginal, but consistent effect in increasing the degree of estimated population structure in the case of *F*_ST_ inference (but not in the cases of STRUCTURE or PC_ST_ analyses).

### 4.1 Impact of filtering on different measures of population structure

PC_ST_ is a nonparametric measure of population structure developed by Linck and Battey (2019) to standardize comparisons of PCAs. In contrast, *F*_ST_ and nucleotide distance (inferred from STRUCTURE) are widely used parametric analyses that have explicit underlying biological assumptions.

Contrary to our hypothesis where we assumed the “Out Any” filter would strengthen the inference of population structure due to the removal of “noisy” loci, we observed little to no effect of this filter on PC_ST_ in any of our simulations. The lack of effect of “Out Any” on PC_ST_ may be explained by the fact that PCA (1) makes no assumptions about the underlying population structure, (2) is nonparametric, or (3) that PC_ST_ is calculated based on only the first 10 principal components, thereby limiting the impact of “noisy” loci on this metric due to the first 10 principal components capturing only the majority of the variation.

In contrast to the PC_ST_ results, for two different parametric methods—STRUCTURE and *F*_ST_—different filtering approaches strongly impacted inferred estimates of population structure. For inferred *F*_ST_ we observe that, with the exception of the extreme population structure scenario (i.e., low migration [*M* = 0.0001]), “Out Any” and “Out Within” tended to lead to inference of marginally higher structure than other filters, in line with our hypothesis that “Out Any” would strengthen inference of population structure. The increase in observed *F*_ST_ in these scenarios (low population structure [*M* = 0.1] to high population structure [*M* = 0.001]) is indicative that filtering using an “Out Any” approach may increase the ability to detect marginal population structure, potentially through increasing the signal to noise ratio of loci reflective of underlying population structure. This pattern was also observed for the “Out Within” filter, albeit in a muted fashion (potentially because this filter removed fewer of the “noisy” loci). However, the “enhanced” ability to detect marginal structure must be viewed with caution as these filters artificially introduced apparent population structure in our analyses of data from a simulated panmictic population.

In contrast, with the exception of marginal population structure (i.e., high migration [*M* = 0.1]), “Out Combo” resulted in reduced inferred population structure in comparison to the other filtering approaches. In the marginal population structure scenario, the migration rate was so high that it is likely that all sampling locations could be considered a single population; therefore, any effects “Out Combo” had on reducing population structure may be outweighed by the increased power to detect HWE deviations as a result of the higher sample size.

In the case of STRUCTURE analyses, we used the average of the nucleotide distance matrix from the STRUCTURE output as a metric to compare analyses, with larger average nucleotide distances between inferred clusters indicative of greater population structure. We found that at lower levels of underlying population structure, the filtering approaches had a greater impact on STRUCTURE results, with “Out Combo” and “Out Any” both leading to marginally higher inferred population structure than the other filters. As population structure increased, these effects were reduced and “Out Any” became comparable with other filters, while “Out Combo” increasingly reduced the average nucleotide distance between populations.

The observation of a reduction in inferred structure associated with filtering across populations (“Out Combo”) can be largely attributed to the introduction of a Wahlund effect, where loci that are informative for population structure (i.e., fixed in one population but not another) are removed due to exhibition of a reduction in heterozygosity as assessed across the total pooled samples. The attribution to a Wahlund effect is further supported by the slopes of regressions of *F*_ST_ against *F*_IS_ (Supporting Information Results, Figures S10 and S11), which show the expected patterns found in Waples (2015). The observation of an increase in inferred population structure (via *F*_ST_) associated with filtering loci that depart from HWE in any population (“Out Any”) could possibly be explained by the selection of loci that conform best to the a priori population groupings. However, in our analyses of simulated panmictic populations, we did not find that the “Out Any” filtering approach introduced artificial structure for STRUCTURE analyses.

Finally, linkage is known to affect population genetic inferences (Waples et al., 2021), something also observed in our data sets, with higher linkage simulations having marginally higher estimates of both *F*_ST_ and PC_ST_. This result, while noticeable, had no noticeable effect on the impacts of different filtering regimes, with relatively consistent results across the two linkage levels.

### 4.2 Comparison to empirical data

The patterns observed in our simulated data were generally also observed in empirical data sets. Specifically, “Out Combo” tended to reduce the inferred amount of population structure for the Plains zebra and New Zealand isopod—both of which have generally high population structure in all other analyses, while for the New Zealand fur seal, no effect of “Out Combo” was observed—consistent with our observations of low population structure in the simulated data sets. However, some discrepancies were observed—for *F*_ST,_ the Plains zebra data set showed reduced inferred population structure in the case of the “Out Any” and “Out Within” filtering approach—contrasting with an increased *F*_ST_ in the simulations with comparable population structure. We further found a significant reduction in STRUCTURE‐inferred average nucleotide distance for the New Zealand isopod when comparing the “Out Any” filter approach with “No Filter” or “Out All”, while our comparable simulations showed no effect of this filter on inferred population structure via STRUCTURE. The discrepancies between the simulated and empirical analyses probably arise from the fact that simulations do not encapsulate the full complexity of real populations. Our simulations do not consider selection, while, for example, the isopod data set was based on morphotypes thought to be under selection (Pearman et al., 2020; Wells & Dale, 2018).

### 4.3 Conclusions and recommendations

We conclude that, despite being a widely used filtering approach, filtering across populations (“Out Combo”) is inappropriate and leads to reduced levels of inferred population structure—especially when population structure is high. Removing loci exhibiting HWE departures in any population (“Out Any”), or removing loci from populations in which they depart from HWE (“Out Within”) can marginally increase the ability to detect population structure in data sets; however, this structure is probably artificially introduced through reliance on an a priori sampling scheme. The impact of removing loci that exhibit departures in every single population (“Out All”) is similar to not filtering at all (“No Filter”). Thus, we suggest that authors conduct thorough exploratory analyses before applying HWE filters, and in general avoid the use of an “Out Combo” filter. Instead, the application of either a “No Filter” or “Out All” regime should be considered. While “Out Any” is more likely to detect population structure, authors should consider the trade‐off between the number of loci lost through application of this filter relative to the information gained. Additionally, authors should consider the risk of introducing spurious structure into their data sets through relying on “Out Any” or “Out Within” filters. Finally, based on lack of convergence in the STRUCTURE runs at extreme population structure, alongside the effects of “Out Any” in increasing population structure, we strongly caution authors to avoid solely relying on sampling schemes as a proxy for population stratification information.

## AUTHOR CONTRIBUTIONS

William S. Pearman and Alana Alexander conceived the study. William S. Pearman, Lara Urban, and Alana Alexander designed the research and analysed the data. William S. Pearman wrote the article with input from both Lara Urban and Alana Alexander.

## CONFLICT OF INTEREST

The authors have no conflicts of interest to declare.

### OPEN RESEARCH BADGES

This article has earned an Open Data, for making publicly available the digitally‐shareable data necessary to reproduce the reported results. The data is available at DOI https://github.com/wpearman1996/HWE_Simulations.

## Supporting information


FIGURE S1–S10

## Data Availability

All R scripts and SLIM scripts are in: https://github.com/wpearman1996/HWE_Simulations References for included datasets are available in the Methods section.
