# Supplementary material for: Commonly used Hardy–Weinberg equilibrium filtering schemes impact population structure inferences using RADseq data
Source: Mol Ecol Resour. 2022 Jun 5;22(7):2599–613. doi: 10.1111/1755-0998.13646 (PMC9541430; doi:10.1111/1755-0998.13646)
Supplement: Supplementary file 1 — FIGURE S1–S10 [file MEN-22-2599-s001.docx]

## Supplementary materials for: Commonly used Hardy-Weinberg equilibrium filtering schemes impact population structure inferences using RADseq data

Authors: William S. Pearman^1,2*^, Lara Urban^2^, Alana Alexander^2^

## Literature search terms

The following search terms were used in the Web of Science to identify papers of interest:

("population genomics" OR "population genetics" OR "genomics" OR "genetics" OR "genetic" OR "genomic") AND ("SNP" OR "GBS" OR "Genotyping-By-Sequencing" OR "Genotyping By Sequencing" OR "RAD" OR "RADseq" OR "ddRAD" OR "RRS" OR "reduced representation sequencing" OR "reduced-representation sequencing" OR "restriction associated" OR "restriction-associated")

Supplementary Figure 1. Diagram of basic underlying simulation, red labels indicate populations that were ‘sampled’ for genetic data, while double sided arrows indicate two-way migration, which varied from M=0.1 to 0.0001.

**
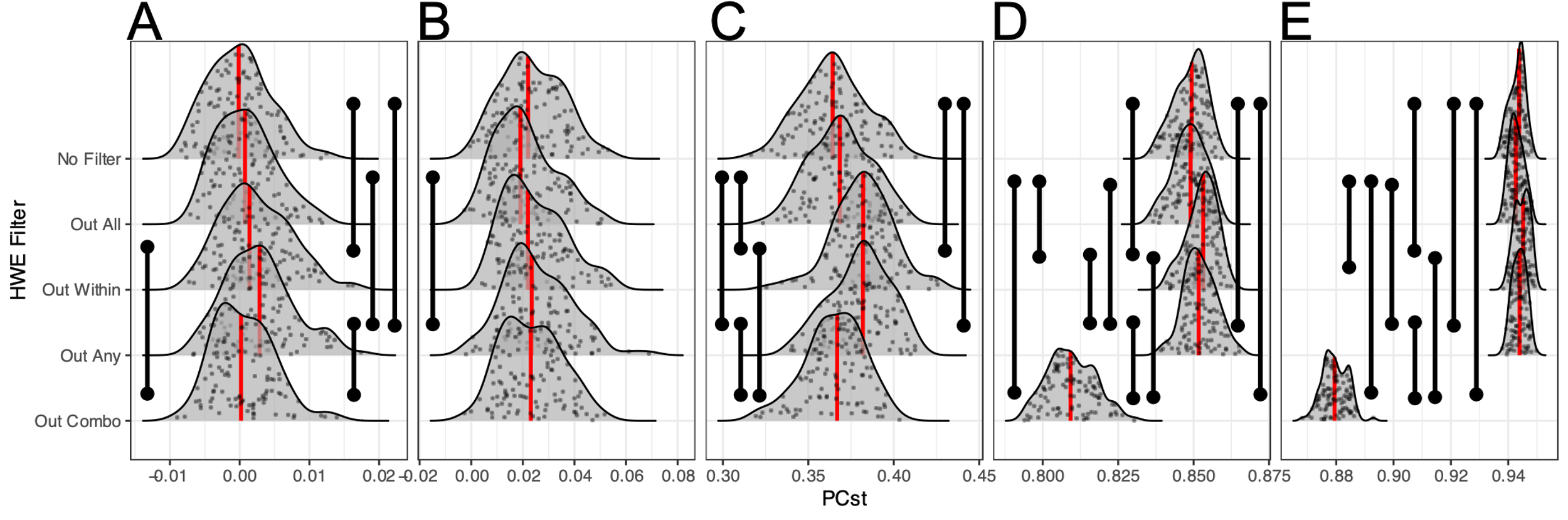
**

Supplementary Figure 2. Distributions of high linkage PC_ST_ across HWE filtering approaches and degrees of population structure. A represents no population structure (single population), B represents marginal population structure (i.e., high migration, M=0.1), C represents low population structure (M=0.01), D represents high population structure (M=0.001), and E represents extreme population structure (i.e., low migration, M=0.0001). Red lines indicate median values, black vertical bars indicate statistically significant comparisons (Mann-Whitney U tests, FDR adjustment).

**
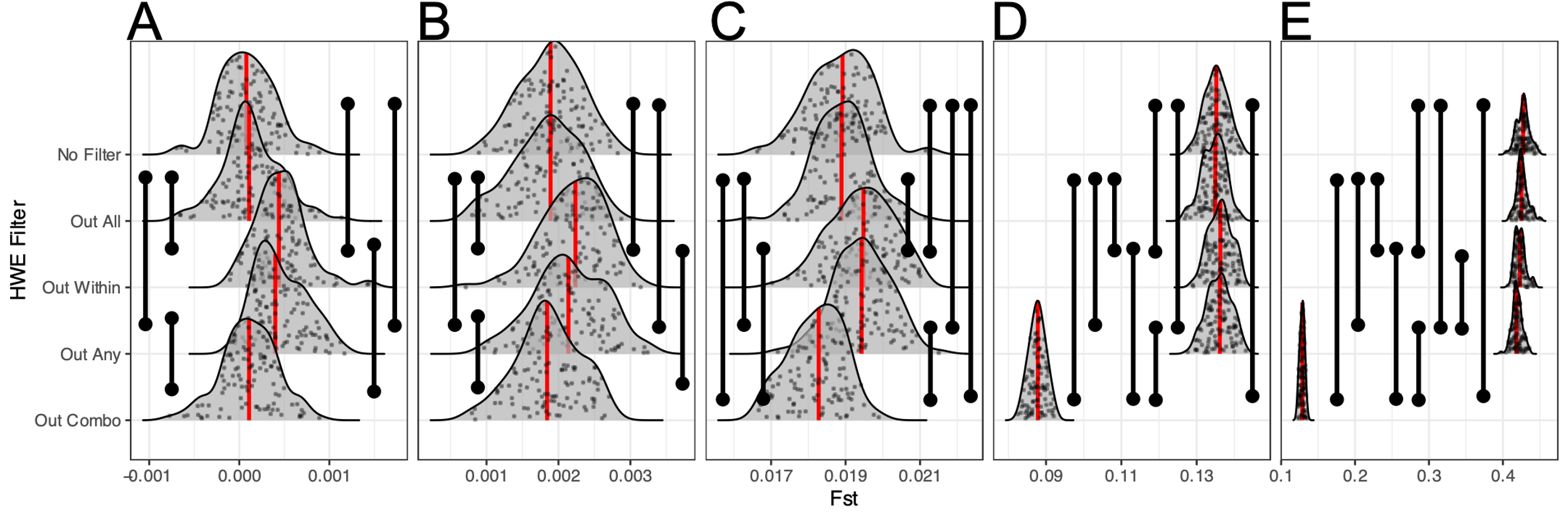
**

Supplementary Figure 3. Distributions of high linkage inferred F_ST_ across HWE filtering approaches and degrees of population structure. A represents no population structure (single panmictic population), B represents marginal population structure (i.e., high migration, M=0.1), C represents low population structure (M=0.01), D is high population structure (M=0.001), and E represents extreme population structure (i.e., low migration, M=0.0001). Red lines indicate median values, black vertical bars indicate statistically significant comparisons (Mann-Whitney U tests, FDR adjustment).

**
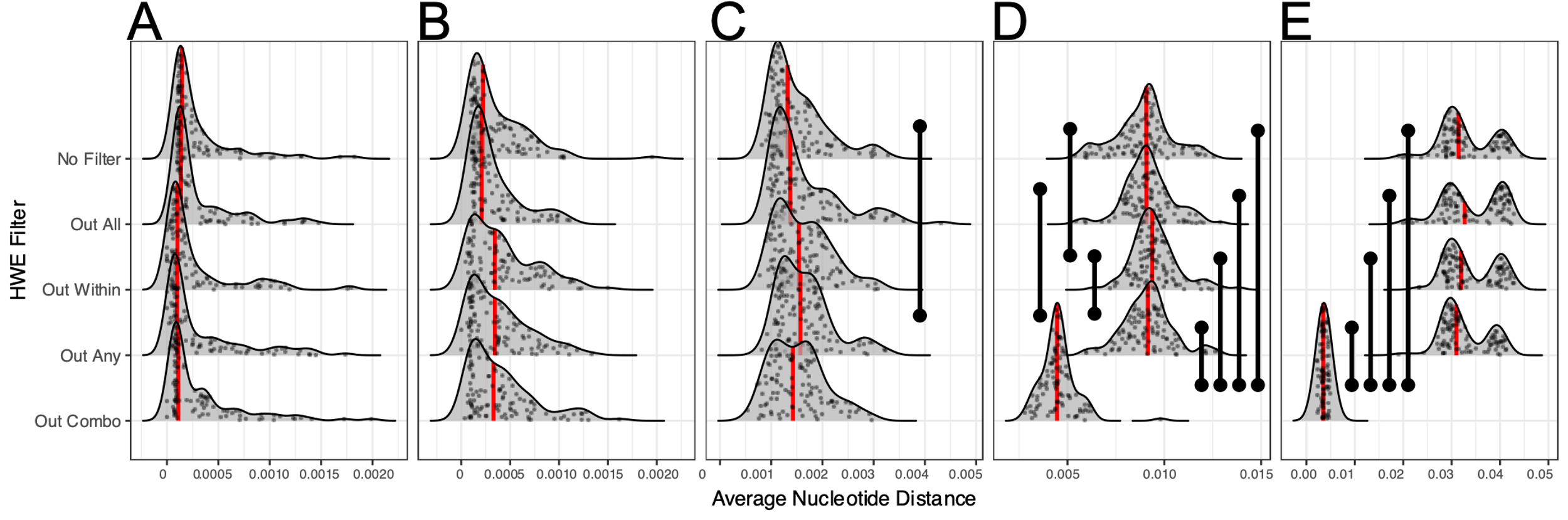
**

Supplementary Figure 4. Distributions of the high linkage average nucleotide distance between inferred population clusters from STRUCTURE, across differing filtering regimes and levels of population structure. A represents a panmictic scenario with no population structure. B represents marginal population structure (i.e., high migration, M=0.1), C represents low population structure (M=0.01), D is high population structure (M=0.001), and E represents extreme population structure (i.e., low migration, M=0.0001). Red lines indicate median values, black vertical bars indicate statistically significant comparisons (Mann-Whitney U tests, FDR adjustment).


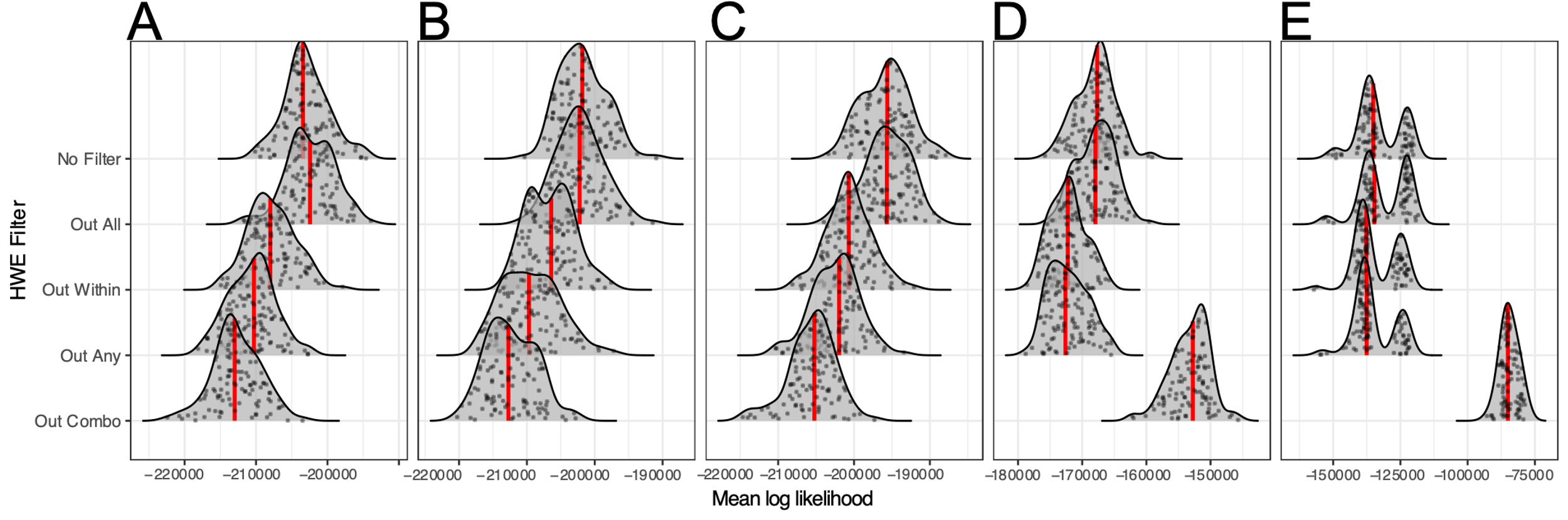


Supplementary Figure 5. Distributions of low linkage mean log likelihood of STRUCTURE runs across HWE filtering approaches and degrees of population structure. A represents no population structure (single panmictic population), B represents marginal population structure (i.e., high migration, M=0.1), C represents low population structure (M=0.01), D represents high population structure (M=0.001), and E represents extreme population structure (i.e., low migration, M=0.0001). Red lines indicate median values.


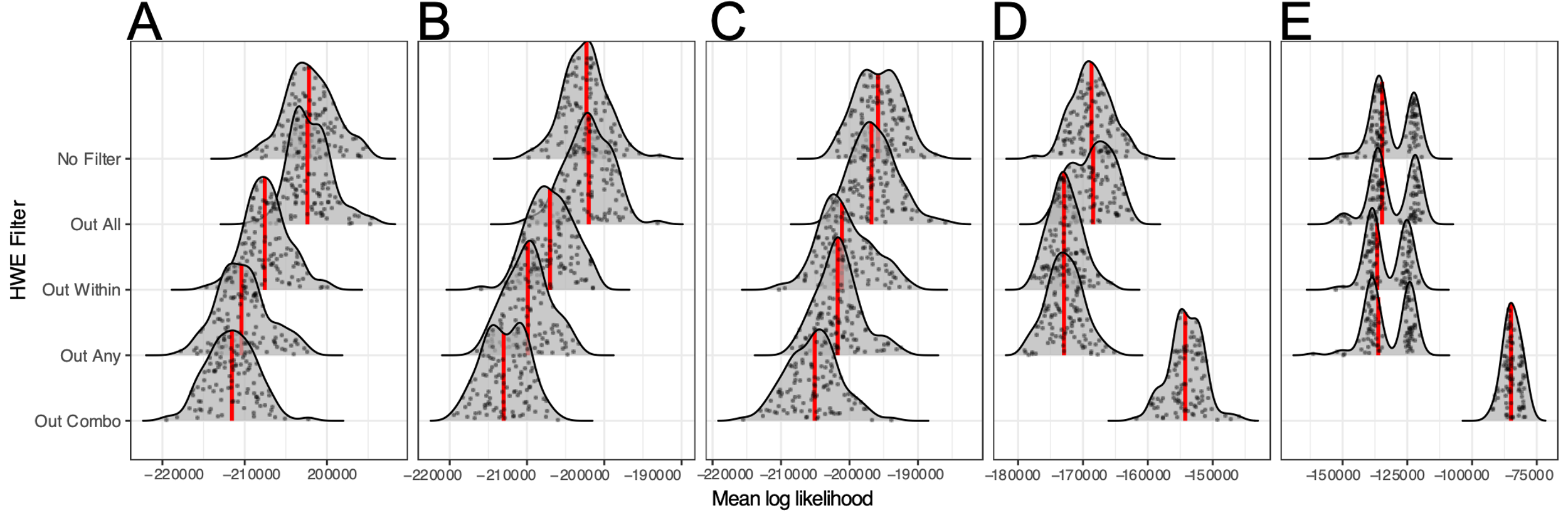
Supplementary Figure 6. Distributions of high linkage mean log likelihood of STRUCTURE runs across HWE filtering approaches and degrees of population structure. A represents no population structure (single population), B represents marginal population structure (i.e., high migration, M=0.1), C represents low population structure (M=0.01), D represents high population structure (M=0.001), and E represents extreme population structure (i.e., low migration, M=0.0001). Red lines indicate median values.


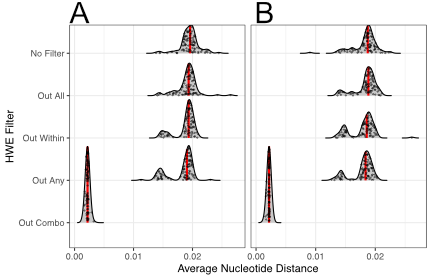


Supplementary Figure 7. Distributions of the average nucleotide distance of STRUCTURE runs for the extreme population scenario. A represents the high linkage scenario, while B represents the low linkage scenario. Red lines indicate median values.


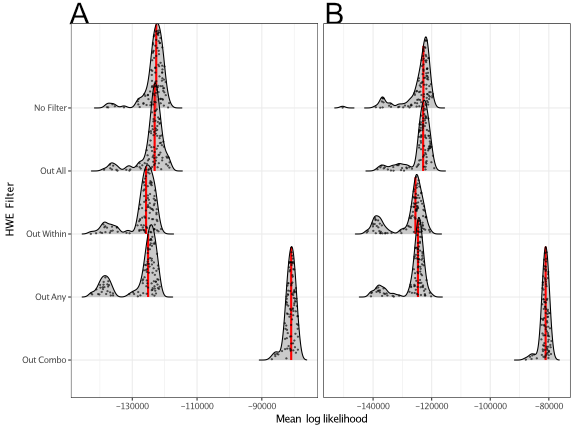


Supplementary Figure 8. Distributions of mean log likelihood of STRUCTURE runs for the extreme population scenario. A represents the high linkage scenario, while B represents the low linkage scenario. Red lines indicate median values.


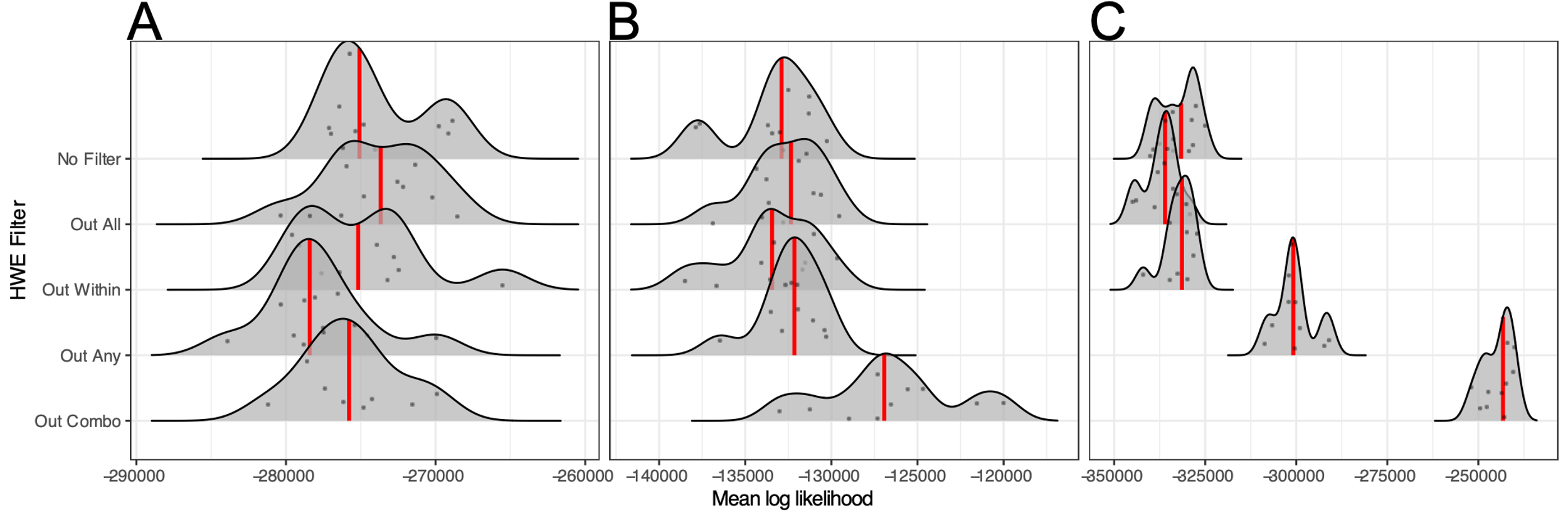


Supplementary Figure 9. Mean log likelihood distributions of STRUCTURE runs for empirical datasets, A represents New Zealand fur seal data (Arctocephalus forsteri), B represents Plains zebra (Equus quagga), and C represents a New Zealand isopod (Isocladus armatus). Red lines indicate the median value for each distribution. Species ordered from low population structure (New Zealand fur seal) to high population structure (isopod). Red lines indicate median values.

## Supplementary Results

We show here that the changes in population structure associated with ‘Out Any’ and ‘Out Within’ are driven by a Wahlund effect. Under a Wahlund effect, there is a predictable relationship between F_ST_ and F_IS_ that should lead to a slope of 1, observed for the ‘Out Any’ and ‘Out Within’ filters (Supp. Fig. 10 & 11). We argue that ‘Out Any’ and ‘Out Within’ filters act to increase the degree of population structure found within a dataset in accordance with the *a priori* population stratification. Thus, in this analysis that pools individuals across sampling locations to examine the presence of a Wahlund effect, we would expect to observe a strong Wahlund effect due to the removal of non-discriminatory loci.


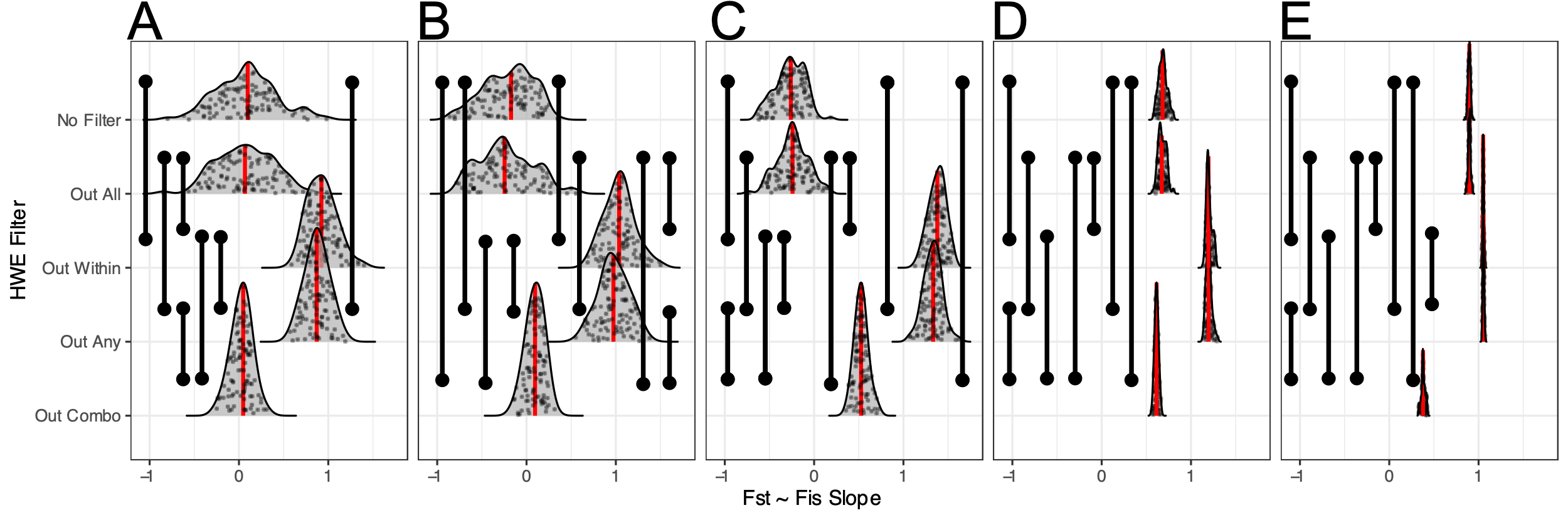


Supplementary Figure 10. Distributions of high linkage slopes between F_ST_ and F_IS_ across HWE filtering approaches and degrees of population structure. A represents no population structure (single population), B represents marginal population structure (i.e., high migration, M=0.1), C represents low population structure (M=0.01), D represents high population structure (M=0.001), and E represents extreme population structure (i.e., low migration, M=0.0001). Red lines indicate median values, black vertical bars indicate statistically significant comparisons (Mann-Whitney U tests, FDR adjustment).


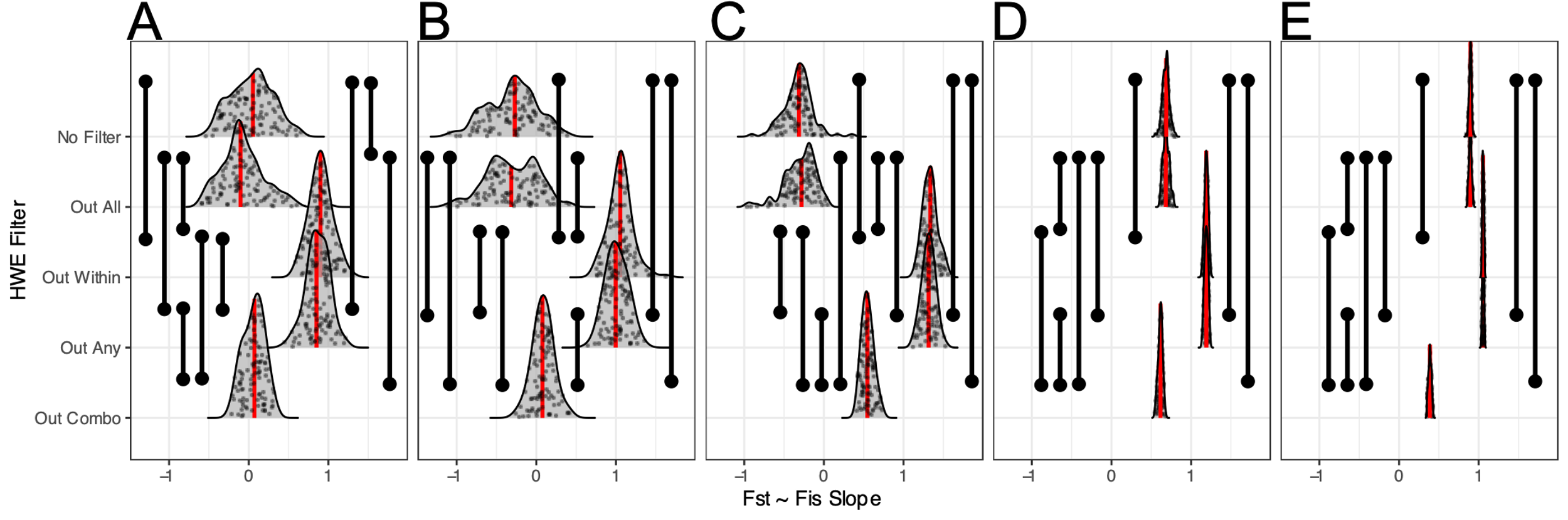


Supplementary Figure 11. Distributions of low linkage slopes between F_ST_ and F_IS_ across HWE filtering approaches and degrees of population structure. A represents no population structure (single population), B represents marginal population structure (i.e., high migration, M=0.1), C represents low population structure (M=0.01), D represents high population structure (M=0.001), and E represents extreme population structure (i.e., low migration, M=0.0001). Red lines indicate median values, black vertical bars indicate statistically significant comparisons (Mann-Whitney U tests, FDR adjustment).
